# Optimization for biogenic microbial synthesis of silver nanoparticles through response surface methodology, characterization, their antimicrobial, antioxidant, and catalytic potential

**DOI:** 10.1038/s41598-020-80805-0

**Published:** 2021-01-12

**Authors:** Saba Ibrahim, Zahoor Ahmad, Muhammad Zeeshan Manzoor, Muhammad Mujahid, Zahra Faheem, Ahmad Adnan

**Affiliations:** 1 Department of Chemistry, University of Engineering and Technology, Lahore, Pakistan; 2 Department of Chemistry, Government College University, Lahore, Pakistan

**Keywords:** Nanoscience and technology, Chemistry, Biochemistry, Biosynthesis, Catalysis, Green chemistry

## Abstract

Silver is a poisonous but precious heavy metal that has widespread application in various biomedical and environmental divisions. Wide-ranging usage of the metal has twisted severe environmental apprehensions. Henceforth there is a cumulative call for the progress of modest, low-cost and, the ecological method for remediation of silver. In the present study, *Bacillus cereus* was isolated from contaminated soil. Various experimental factors like the amount of AgNO_3_, inoculum size, temperature, time, and pH were improved by using central composite design (CCD) grounded on response surface methodology (RSM). Optimized values for AgNO_3_ (1 mM) 10 ml, inoculum size (*Bacillus cereus*) 8.7 ml, temperature 48.5 °C, time 69 h, and pH 9 showed in the form of optimized ramps. The formed nanoparticles stayed characterized by UV–visible spectrophotometer, Scanning Electron Microscopy, Fourier transform infra-red spectrometry, particle size analyzer, and X-ray diffraction. The particle size ranges from 5 to 7.06 nm with spherical form. The antimicrobial effectiveness of synthesized nanoparticles was tested contrary to five multidrug resistant microbial strains, *Staphylococcus epidermidis, Staphylococcus aureus*, *Escherichia coli, Salmonella enterica, Porteus mirabilis* by disc diffusion method. The minimum inhibitory concentrations and minimum lethal concentrations were detected by the broth macro dilution method. 2,2-diphenyl-1-picrylhydrazyl-hydrate (DPPH) was used to check the free radical scavenging ability of biogenic silver nanoparticles. Similarly, anti-radical activity was checked by 2,2′-Azino-Bis-3-Ethylbenzothiazoline-6-Sulfonic Acid (ABTS) with varying time intervals. Catalytic potential of biosynthesized silver nanoparticles was also investigated.

## Introduction

Nanotechnology is beholding as congregating expertise of the recent times due to its structural constancy overages and its roles concerning every arena of science. Currently, many physical and chemical methods are there but generally, they are costly and hazardous for living things and the environment^1^. In the field of nanochemistry, metal nanoparticles (MNPs) have grabbed imperative consideration in extensive applications due to their exclusive physicochemical properties characteristic to the nanoscale^2^. The approaches of synthesis of metal nanoparticles are as varied as the nature of various metals. The approaches have been classified in the top-down mechanism^3^ and a bottom-up mechanism^4^. For the synthesis of nanomaterials, its approach to natural machinery to achieve an eco-friendly nanostructured material for more remediation^5,6^. Engaging any biological element such as plant, yeast, bacteria, and fungi is always an excellent choice for an environmentally responsive method that is accredited to be green synthesis^7,8^. Besides the support of the ecosystem, green synthesis has been found to be helpful in controlling the size and shape of nanoparticles of our interest^9^. Recently the usage of microbes has been highlighted in living systems for the synthesis of metallic NPs^10^. Several microbes have exceptional potential for bioremediation of noxious metals and their translation into more docile forms, and this makes them necessary aspirants for living systems. Different microorganisms practice extracellular or intracellular routes for biosynthesis of NPs. Biotic synthesis of metallic NPs can be achieved by using either complete cell masses of bacteria, fungi, and algae or using cell extracts or culture supernatant of microorganisms^11,12^. Microbial synthesis of metal nanoparticles requires different physicochemical and biological parameters which help in acquiring metal nanoparticles with controlled size, shape, and dispersity^13^. In recent years, nanostructured materials verified to have high potential to attain definite procedures particularly in biological and pharmaceutical applications which fascinate a boundless impact of attention^14^. Over the past periods, silver has been proved to be a harmless inorganic antibacterial agent that is non-toxic and well recognized for the assassination of about 650 kinds of diseases producing microbes^15–17^. Silver has been labeled as energetic for its capability to exercise potential for a varied variety of biological applications such as anti-fungal agents, anti-bacterial agents, avoiding contagions, healing wounds, and anti-inflammatory even at low concentrations^18,19^. Silver ions are involved in the devising of dental resins, bone adhesive, ion exchange fibers, and medical tools coating^20^. Recent studies have shown that silver nanoparticles show strong inhibition and assist as a defensive barrier for most of the pathogens^21,22^. Apart from biomedical tenders^23^, silver nanoparticles find extensive applications in catalysis, optical properties, wastewater treatment, plant growth, and crop production^24–27^.

Motivation behind this research work is to develop a simple, low-cost, eco-friendly method for microbial synthesis of AgNPs. The effect of various experimental parameters like temperature, pH, time, amount of silver nitrate, and inoculum size was also optimized through response surface methodology (RSM). For experimental design, RSM is a useful technique to optimize experimental parameters and AgNPs preparations by minimizing the number of trials and errors^28,29^. This represents a comprehensive investigation of various experimental variables in the microbial synthesis of AgNPs using central composite design (CCD). The biosynthesized AgNPs were characterized through various techniques and were studied for antimicrobial, antioxidant, and catalytic applications.

The usage of conservative ways of optimization of biogenic synthesis of silver nanoparticle by changing one factor at a time like temperature, time, pH, metal concentration, and inoculum size by keeping other factors constant is difficult, dull, cost-inefficient, long, and likewise incapable to govern reciprocated interactive properties of diverse factors. Response surface methodology (RSM) is the greatest extensively modified arithmetical and mathematical software employed for the optimization of multiple factors fluctuating simultaneously. RSM can offer thorough intuition of straight, curvilinear, and pairwise combined effects of variables on the response^30^. Multivariate practices are available for experimental designs, which are Box–Behnken Design (BBD), Central Composite Design (CCD), and Doehlert Matrix (DM). In terms of efficiency of design, CCD is more efficient as compared to BBD and DM^31^.

The recent study revealed that the biological production of silver nanoparticles from *Bacillus cereus* was carried out. The parameters were optimized by using statistical tool RSM and were categorized using simple systematic techniques of UV–visible spectrophotometer, scanning electron microscopy (SEM), Furrier transform infra-red (FTIR) spectroscopy, particle size analyzer, and X-ray diffraction (XRD). Antimicrobial action of biosynthesized silver nanoparticles was inspected with five species of multidrug-resistant microbes. In these strains, two gram-positive bacteria, *Staphylococcus epidermidis, Staphylococcus aureus*, and three gram negative bacteria as *Escherichia coli, Salmonella enterica, Porteus mirabilis* by disc diffusion method using streptomycin as a standard antibacterial agent. During this study, the minimum inhibitory concentrations (MICs) and minimum lethal concentrations (MLCs) were also spotted by the broth macro dilution method. Antioxidant potential of biosynthesized silver nanoparticles was estimated by scavenging free radicles of 2,2-diphenyl-1-picrylhydrazyl-hydrate (DPPH) and similarly it was also checked by 2, 2′-Azino-Bis-3-Ethylbenzothiazoline-6-Sulfonic Acid (ABTS) with varying time intervals. The scavenging action was monitored by UV–vis spectrophotometer. The catalytic potential of silver nanoparticles was studied for the degradation of methyl orange in the industrial wastewater.

## Results and discussion

**Figure 1:**
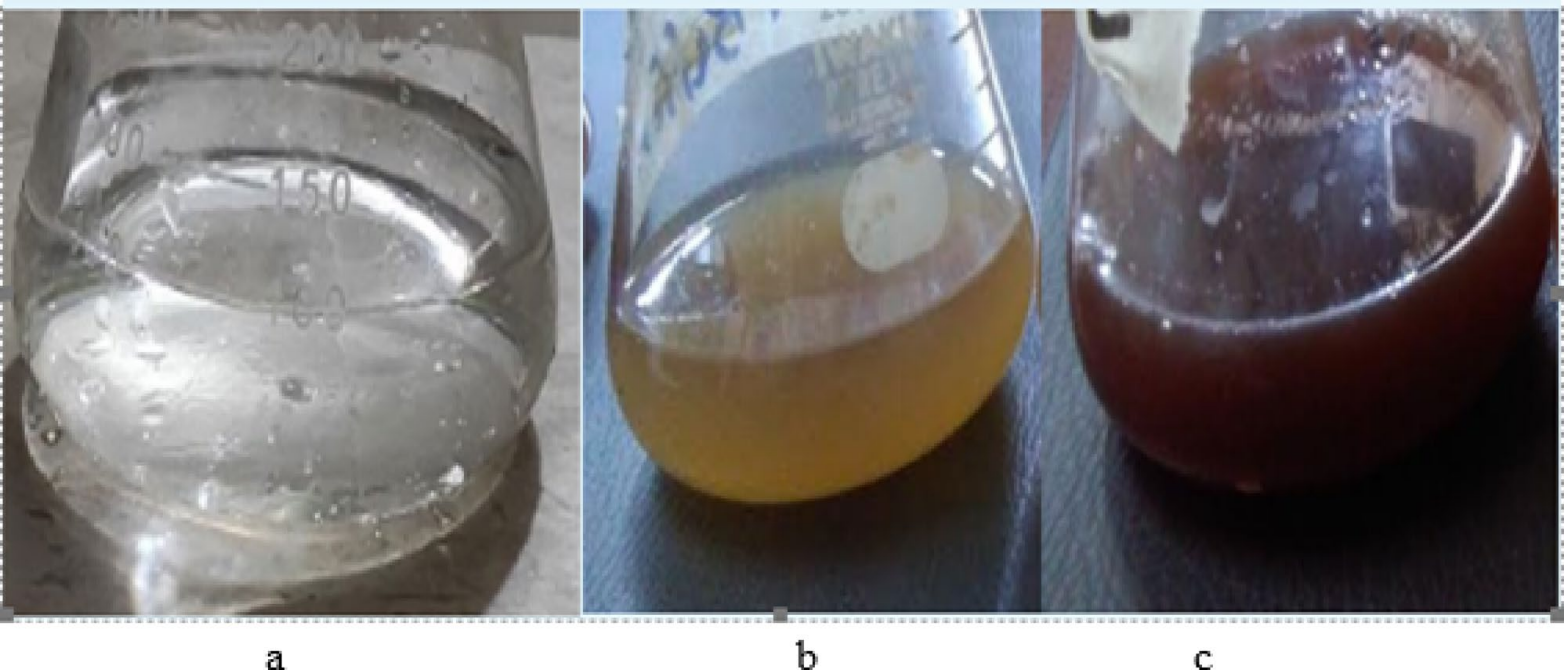
(**a**–**c**) Visible observation for green synthesis of silver nanoparticles by *Bacillus cereus* (**a** = Silver nitrate solution, **b** = silver nitrate and *Bacillus cereus*, **c** = synthesized silver nanoparticles).

Visually the biogenic preparation of silver nanoparticles was retrieved through the variation in color of the reaction combination to a dark brown but no color change was observed in control (without AgNO_3_ salt solution) as in Fig. 1. To study the distinct and joint effects of different parameters like metal concentration (ml of 1 mM), inoculum size (*Bacillus cereus*), temperature, time, and pH Central Composite Design (CCD) per 26 experiments was established. For this purpose, Response Surface Methodology Design-Expert-12 software was used. The central composite design consisted of eleven full factorial, ten axial, and five center points positioned at both extreme and vital points (Table 2) correspondingly. Regression examination of the data points studied the distinct and collaborative effects of variables on the biosynthesis of silver nanoparticles and a second-order polynomial equation stood settled. The low (− 1), central (0), and high (+ 1) values of variables like AgNO_3_ concentration (ml of 1 mM), inoculum size, temperature, time, and pH were coded as A, B, C, D, and E respectively (Table 1) whereas absorbance at 430 nm indicated the experimental response of all the 26 different groupings of experimental factors (Table 2).

**Table 1:** Experimental values and levels of independent variables.

Independent variables	Symbols	Low (− 1)	Centre (0)	High (+ 1)
AgNO_3_ 1 mM (ml)	A	1	5.5	10
Inoculum size (ml)	B	1	5.5	10
Incubation temperature (°C)	C	25	37.5	50
Time (h)	D	12	42	72
pH (Mol/L of H^+^ ions)	E	5	7	9

**Table 2:** The central composite design for the experiment.

Std	Run	Space type	Factor 1	Factor 2	Factor 3	Factor 4	Factor 5	Response 1
			A: AgNO_3_ (1 mM)	B: inoculum size	C: temperature	D: time	E: pH	Absorbance
			ml	ml	°C	h	Mol/L of H + ions	nm
13	1	Axial	10	5.5	37.5	42	7	1.32
22	2	Center	5.5	5.5	37.5	42	7	2.16
14	3	Axial	5.5	1	37.5	42	7	0.25
6	4	Factorial	10	1	25	72	9	1.63
2	5	Factorial	10	1	50	72	5	1.5
9	6	Factorial	10	1	50	12	9	0.98
16	7	Axial	5.5	5.5	25	42	7	1.4
12	8	Axial	1	5.5	37.5	42	7	2.61
26	9	Center	5.5	5.5	37.5	42	7	1.62
18	10	Axial	5.5	5.5	37.5	12	7	2.28
20	11	Axial	5.5	5.5	37.5	42	5	2.73
4	12	Factorial	10	10	50	12	5	1.61
3	13	Factorial	1	10	50	12	9	3.29
1	14	Factorial	10	10	25	72	5	0.57
21	15	Axial	5.5	5.5	37.5	42	9	1.51
5	16	Factorial	10	10	25	12	9	0.18
8	17	Factorial	1	10	25	72	9	0.62
25	18	Center	5.5	5.5	37.5	42	7	1.66
7	19	Factorial	1	1	50	72	9	2.77
19	20	Axial	5.5	5.5	37.5	72	7	1.43
24	21	Center	5.5	5.5	37.5	42	7	1.77
23	22	Center	5.5	5.5	37.5	42	7	1.62
10	23	Factorial	1	10	50	72	5	2.82
11	24	Factorial	1	1	25	12	5	2.58
15	25	Axial	5.5	10	37.5	42	7	2.63
17	26	Axial	5.5	5.5	50	42	7	3.14

**Figure 2:**
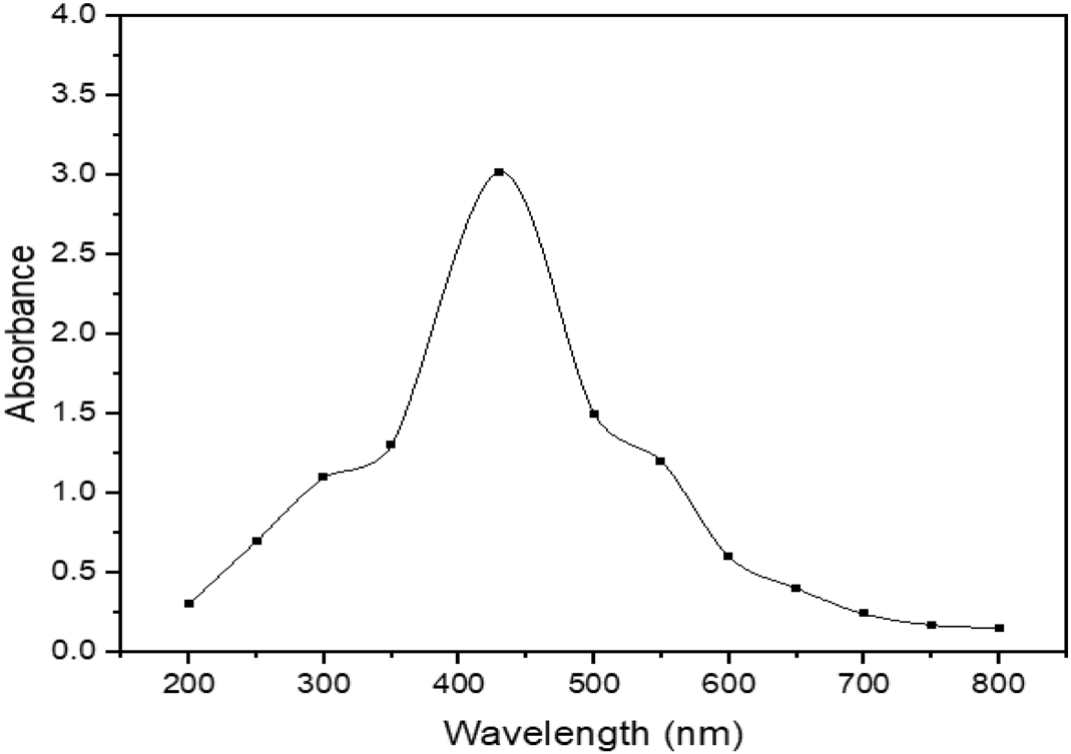
The UV–visible spectrum of biosynthesized silver nanoparticles by using *Bacillus cereus.*

The color change from transparent to light yellow and then dark brown is the visual signal for the conversion of silver nitrate into silver nanoparticles as shown in (Fig. 1a–c), however, it was further confirmed by the UV–visible spectrophotometer. The UV–Visible spectrum was predictable on the UV-1700 pharmaspac UV–Visible spectrophotometer functioned at a resolution of 1 nm. The wavelength range was 400–800 nm. A robust surface plasmon response (SPR) peak was observed at 430 nm (Fig. 2). The UV–visible absorption peak in the range of 400 nm to 500 nm is the characteristic surface plasmon peak of silver nanoparticles^32^. Similarly, morphological information of biosynthesized silver nanoparticles can also be obtained by the analysis of UV–visible spectra. In this study, the silver nanoparticles produced are isotropic and spherical due to the appearance of a single surface plasmon response peak^33^. The dark brown solution produced was centrifuged at 5000 rpm for 10 min and air-dried to get the powder of silver nanoparticles.

**Figure 3:**
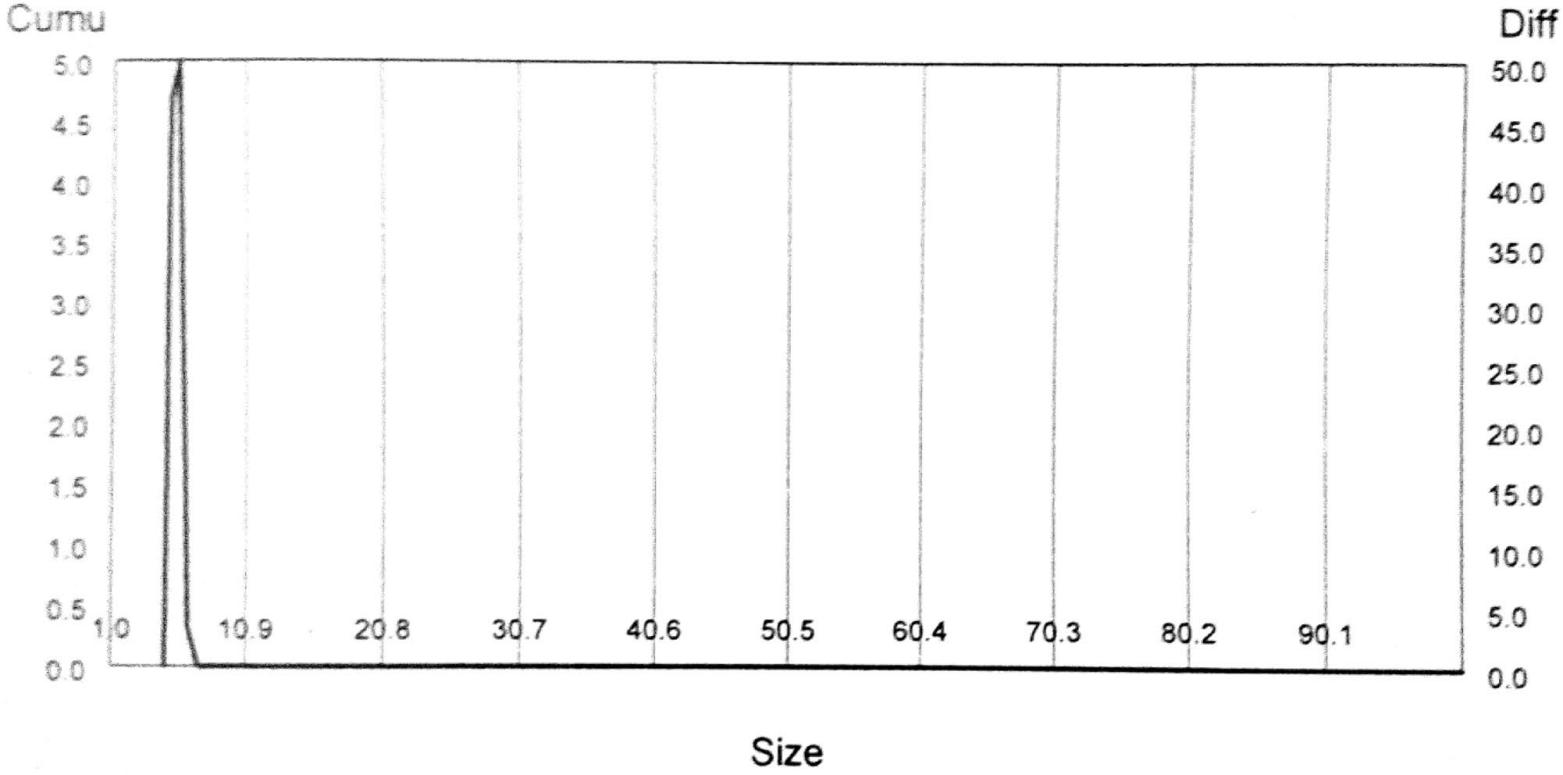
Particle size distribution of biosynthesized silver nanoparticles.

The particle size of silver nanoparticles was measured through Dynamic Light Scattering (DLS) method (BT-90 nanoparticle size analyzer instrument). Intensity of light scattered due to the movement of particles (Brownian motion) was measured. It examines the particles perpendicular to the light source. To calculate the hydrodynamic diameter of biosynthesized particles Stokes–Einstein equation was employed^34^. Solution of biosynthesized silver nanoparticles was prepared by dissolving 1 mg in 5 ml of deionized water. For homogenization of the prepared solution, it was ultrasonicated for 30minutes. After that 10 µl of the above solution is transported to 1 ml cuvette, filled the remaining portion with deionized water, and placed in particle size analyzer. It showed the D_50_ value 5.65 nm, and D (4.3) value 5.59 nm with 358.78 m^2^/g surface area (Fig. 3).

**Figure 4:**
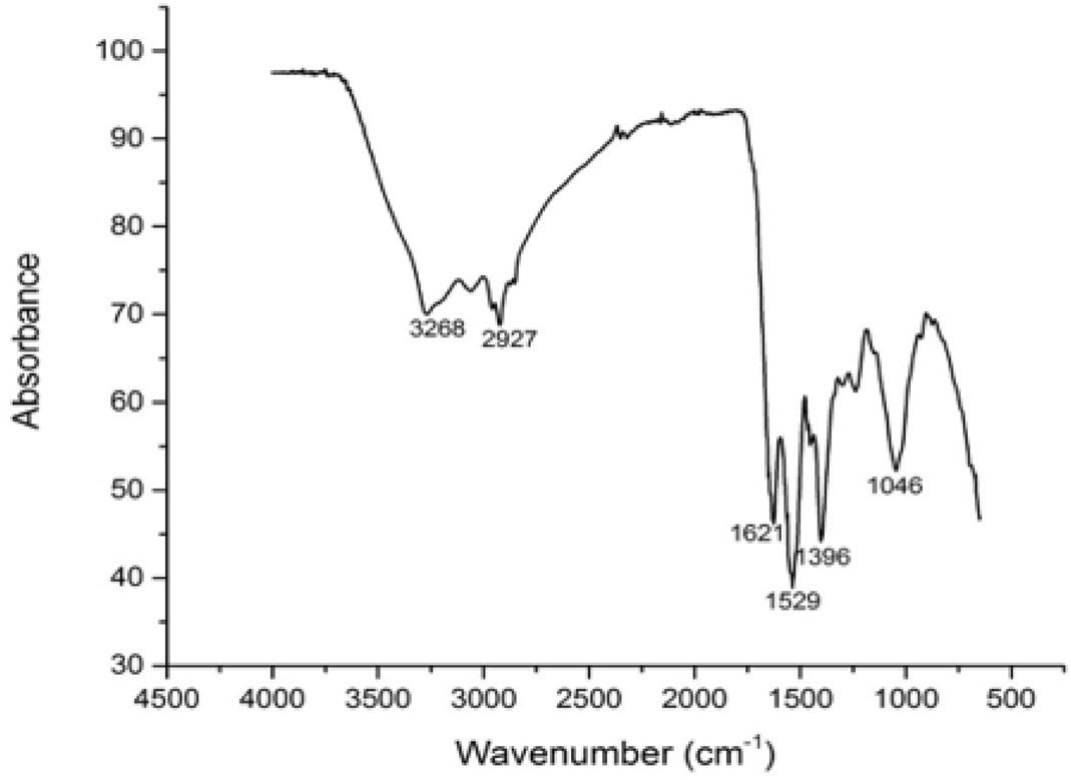
FTIR spectra of biosynthesized silver nanoparticles by *Bacillus cereus*.

To find out the practical assemblies and their probable corporation in the biosynthesized silver nanoparticles, the dry product was ground with potassium bromide and the range was plotted by using furrier transform infra-red spectroscopy VERTEX 70, Japan. Furrier transform infra-red spectroscopy was used to locate the capping of biosynthesized silver nanoparticles. FTIR ranges perused from 500 to 4500 cm^−1^ showed the incidence of functional assemblies of biomolecules. FTIR spectra of biosynthesized silver nanoparticles showed six major peaks at 1046 cm^−1^, 1396 cm^−1^, 1529 cm^−1^, 1621 cm^−1^, 2927 cm^−1^, and 3268 cm^−1^ (Fig. 4). FTIR investigation was made to find out the connections between silver and biologically lively compounds that are responsible for the creation and constancy of nanoparticles as capping agents^35^. The carboxylic acid group derived with an amine in the amino acid of proteins designates in the infrared region of the range. The occurrence of proteins are specified by such a region of the spectrum. The connections among nanoparticles and proteins were due to the unrestricted amine group and at the same time can also be through negative carboxyl groups of enzymes. It was obvious that proteins or enzymes released from the bacterial cell are in authority for translation and stabilization of silver nanoparticles.

**Figure 5:**
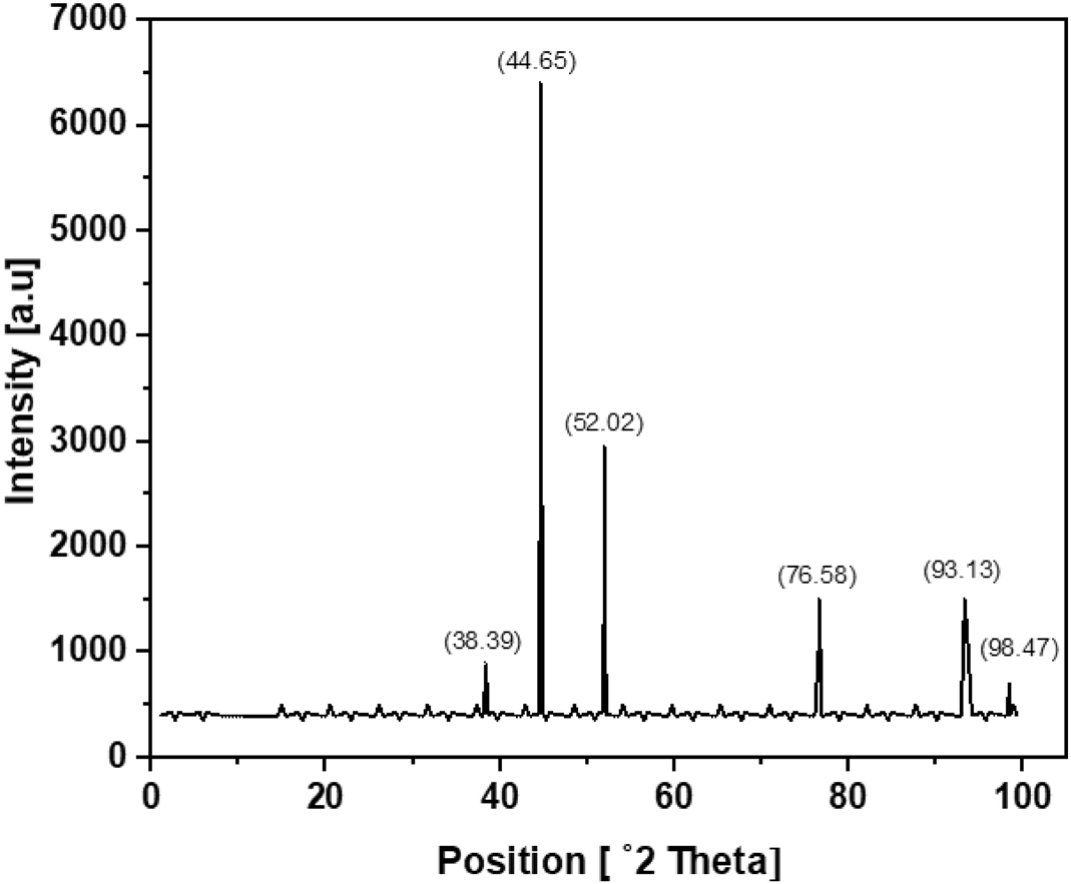
X-ray diffraction pattern of silver nanoparticles. Silver nanoparticles were synthesized from 1 mM silver nitrate treated with *Bacillus cereus*. The samples were sonicated, air-dried and the XRD pattern was observed.

To investigate the nature of prepared nanoparticles, X-ray diffraction (XRD) of the product was worked by Philips PANalytical X’Pert Powder (Park system, Korea). The peaks expressed were allocated to consistent diffraction signals. The XRD outline of biosynthesized silver nanoparticles displayed four robust peaks in the whole spectrum of 2θ values extending from 20 to 80 (Fig. 5). These reflections were indexed as (111), (200), (220), and (311). A comparison of our XRD pattern with slandered (JCPDS Card No 87-0597) had established the synthesis of silver nanoparticles.

A field emission Scanning Electron Microscope (SEM) study was completed in an instrument FEI Nova 450 NanoSEM. SEM provides information about exterior morphology, structure, chemical composition, and alignment of the sample. SEM provides high-resolution pictures of samples by focusing on the primary electron beam and detecting secondary or backscattered electron signals.

**Figure 6:**
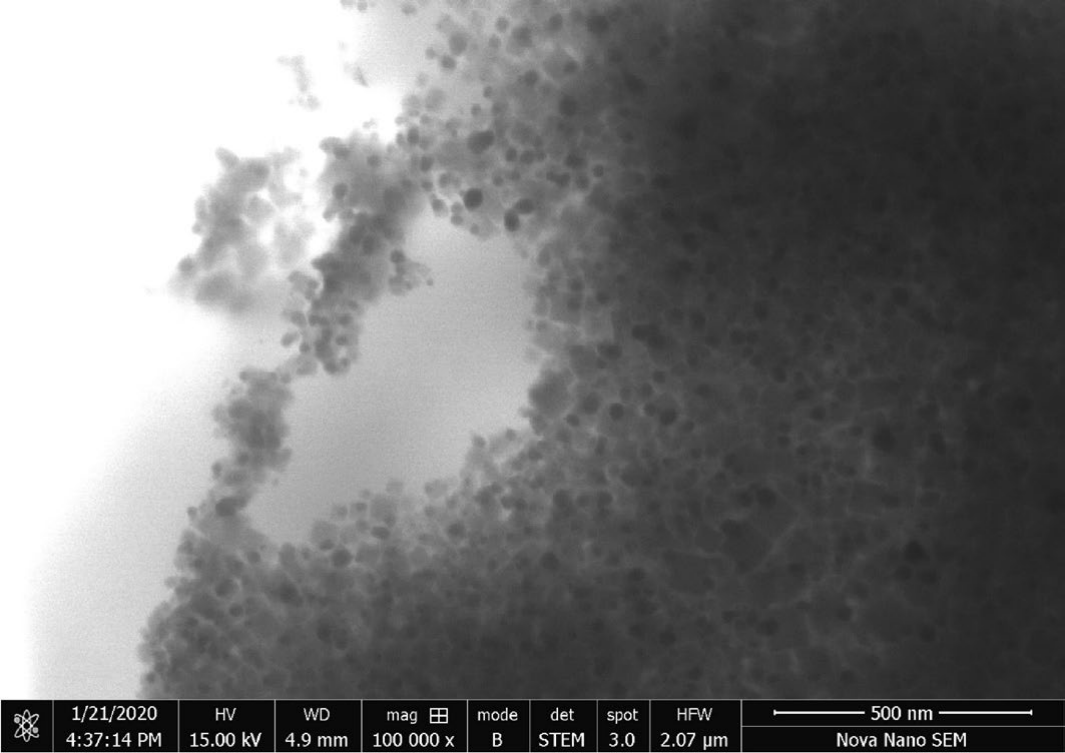
SEM image of produced silver nanoparticles.

SEM micrograph of synthesized silver nanoparticles is shown in (Fig. 6).

The P-values regulate the meaning of direct, quadratic contact of coefficients. A P-value of less than 0.0500 designates model terms are considerable. In this situation, A, B, C, E, AB, AD, AE, BC, BD, CE, DE, B^2^ are noteworthy prototypical standings. Values greater than 0.1000 indicate the terms of design are not important. The changeability in predicted and real responses and fit statistics of the established design was confirmed by coefficient R^2^. The value of intended R^2^ 0.9825 showed that 98.25% variation is due to independent variables in the biosynthesis of silver nanoparticles. The model F-value of 14.01 suggests the model is significant. There is only a 0.41% coincidental that an F-value this great could rise due to noise. The non-significant f-value of 2.25 and p-value 0.2083 infers that the nominated regression design is significant. There is a 20.83% chance that a f-value this large could arise due to noise. Predicted R^2^ suggests that the total mean maybe an improved analyst of response. Adequate precision dealing with the signal to noise fraction. A fraction larger than 4 is wanted. the ratio of 13.597 specifies a satisfactory signal. This model can be used to navigate the design space. The analysis of variance (ANOVA) and fit statistics of the quadratic model to test its significance is described in Tables 3 and 4.

**Table 3:** ANOVA for quadradic model.

Source	Sum of squares	df	Mean square	F-value	p-value	
Model	18.30	20	0.9152	14.01	0.0041	Significant
A-AgNO_3_ (1 nM)	0.8320	1	0.8320	12.74	0.0161	
B-inoculum size	2.83	1	2.83	43.36	0.0012	
C-temperature	1.51	1	1.51	23.18	0.0048	
D-time	0.3612	1	0.3612	5.53	0.0654	
E-ph	0.7442	1	0.7442	11.39	0.0198	
AB	1.01	1	1.01	15.52	0.0110	
AC	0.0212	1	0.0212	0.3250	0.5933	
AD	1.50	1	1.50	22.95	0.0049	
AE	1.38	1	1.38	21.17	0.0058	
BC	1.46	1	1.46	22.30	0.0052	
BD	0.6185	1	0.6185	9.47	0.0276	
BE	0.3046	1	0.3046	4.66	0.0832	
CD	0.2122	1	0.2122	3.25	0.1313	
CE	0.4331	1	0.4331	6.63	0.0497	
DE	2.44	1	2.44	37.35	0.0017	
A^2^	0.0005	1	0.0005	0.0073	0.9352	
B^2^	0.6393	1	0.6393	9.79	0.0260	
C^2^	0.2491	1	0.2491	3.81	0.1083	
D^2^	0.0226	1	0.0226	0.3457	0.5821	
E^2^	0.0699	1	0.0699	1.07	0.3484	
Residual	0.3266	5	0.0653			
Lack of fit	0.1175	1	0.1175	2.25	0.2083	Not significant
Pure error	0.2091	4	0.0523			
Cor total	18.63	25

**Table 4:** Fit statistics of the quadratic model.

Std. dev	0.2556	R^2^	0.9825
Mean	1.80	Adjusted R^2^	0.9124
C.V. %	14.23	Predicted R^2^	− 54.4223
		Adeq precision	13.5972

**Figure 7:**
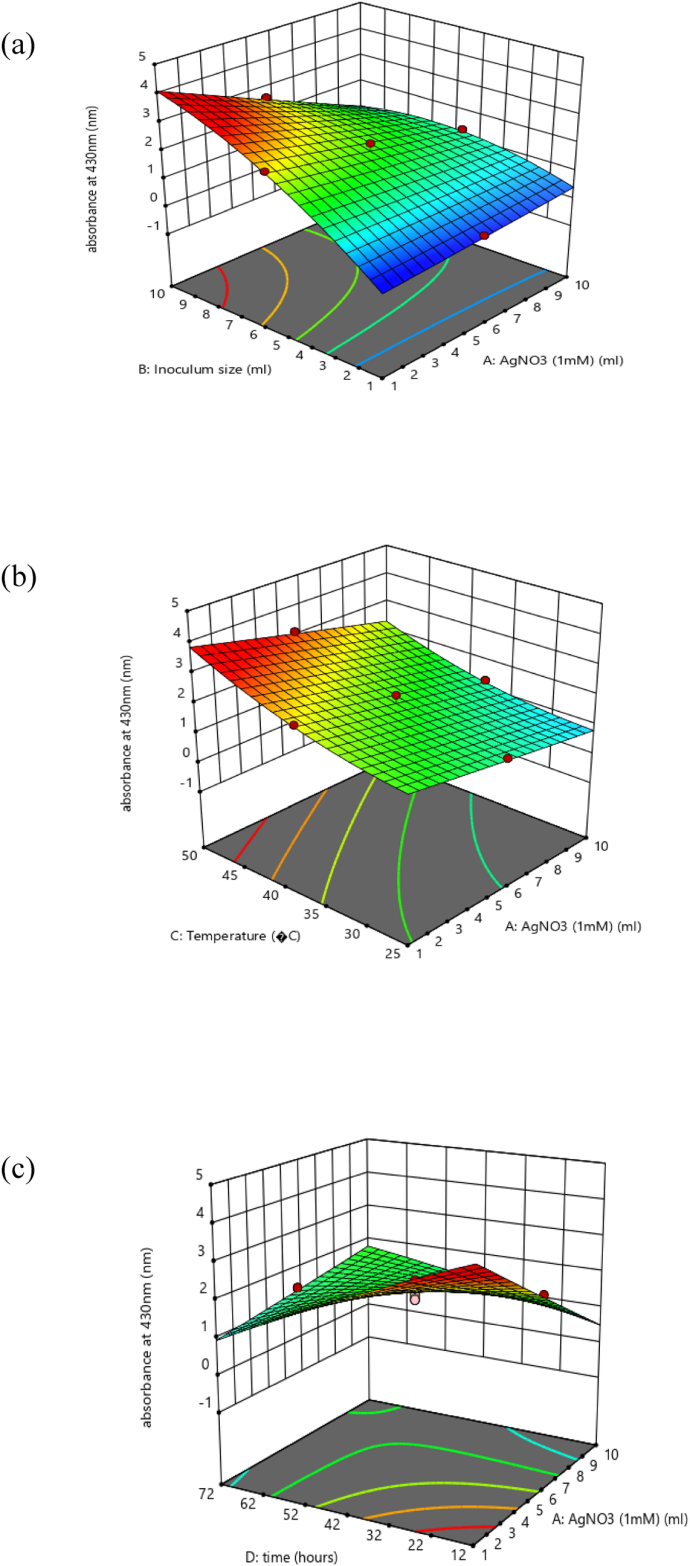

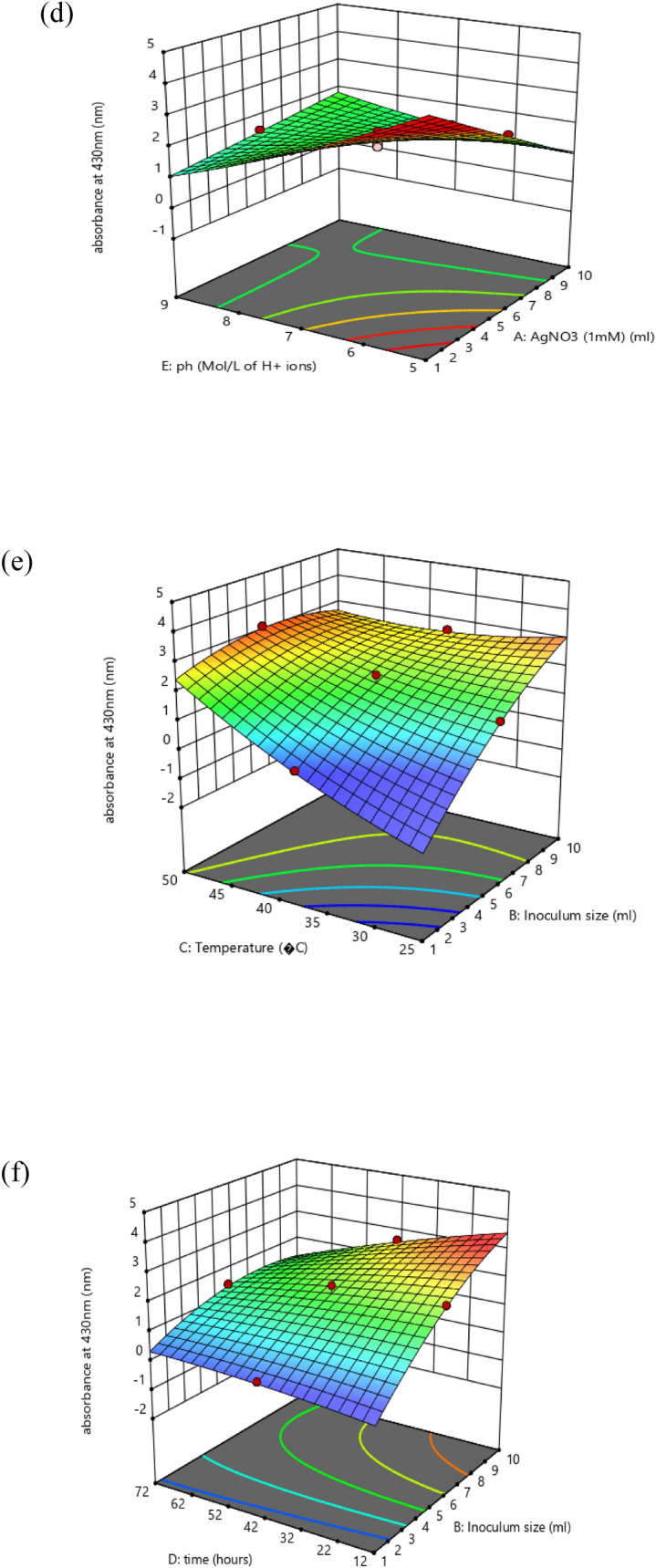

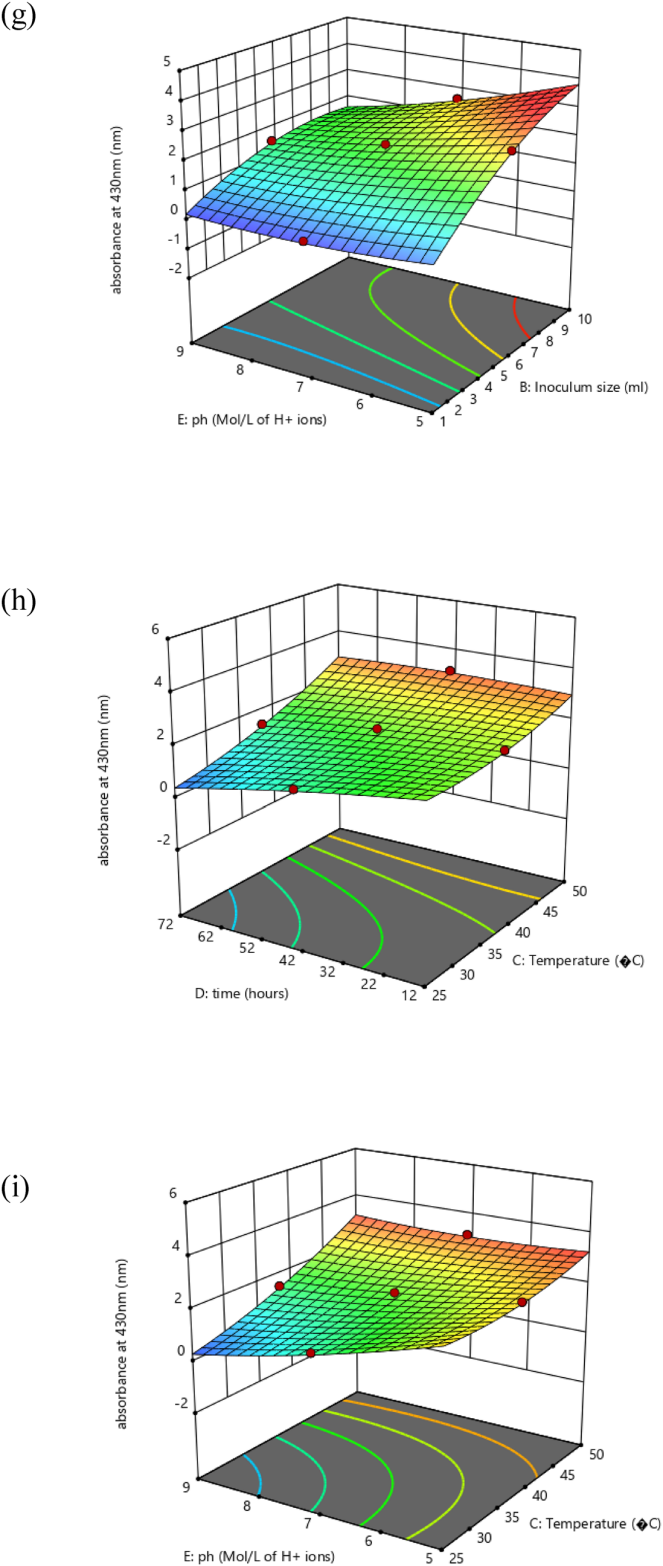

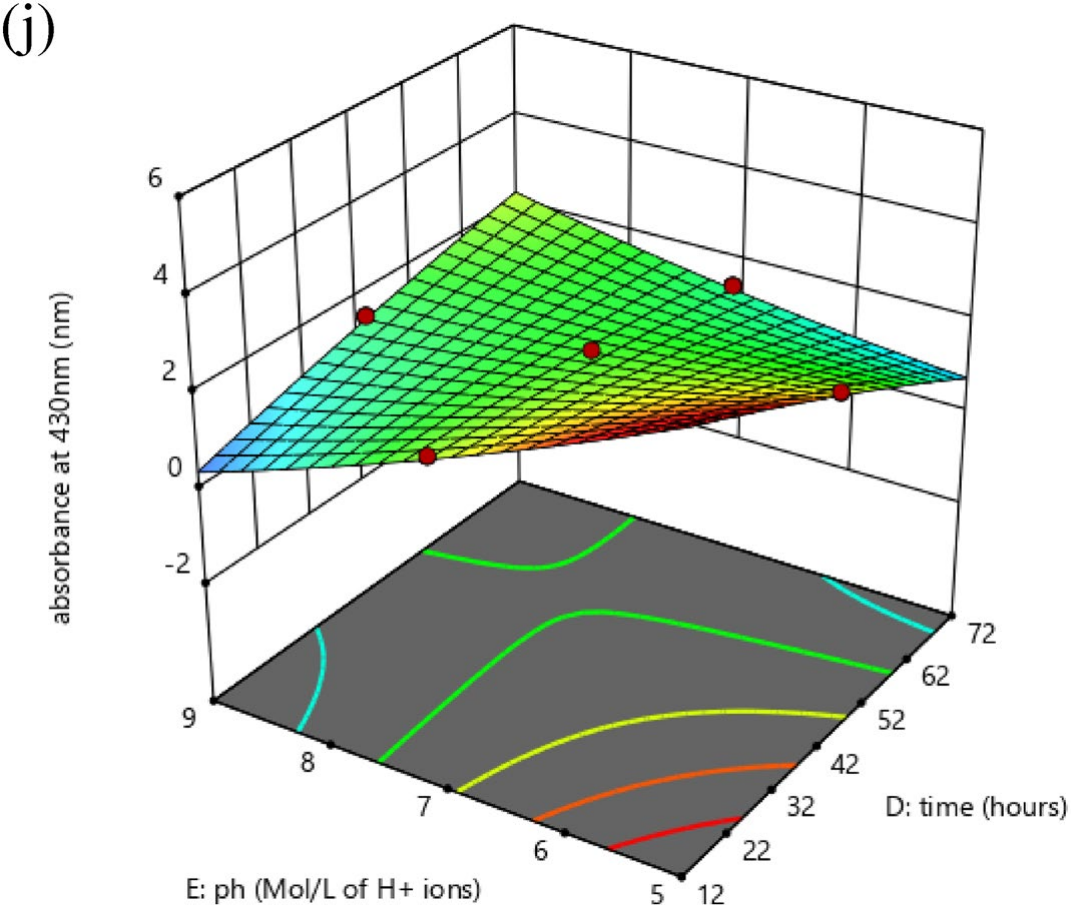
Three dimensional graphics for response surface optimization by plotting absorbance versus (**a**) silver nitrate (ml) and inoculum size (ml), (**b**) silver nitrate (ml) and temperature (°C), (**c**) silver nitrate (ml) and time (hours), (**d**) silver nitrate (ml) and pH, (**e**) inoculum size (ml) and temperature (°C), (**f**) inoculum size (ml) and time (hours), (**g**) inoculum size (ml) and pH, (**h**) temperature (°C) and time (h), (**i**) temperature (°C) and pH, (**j**) time (h) and pH.

**Figure 8:**
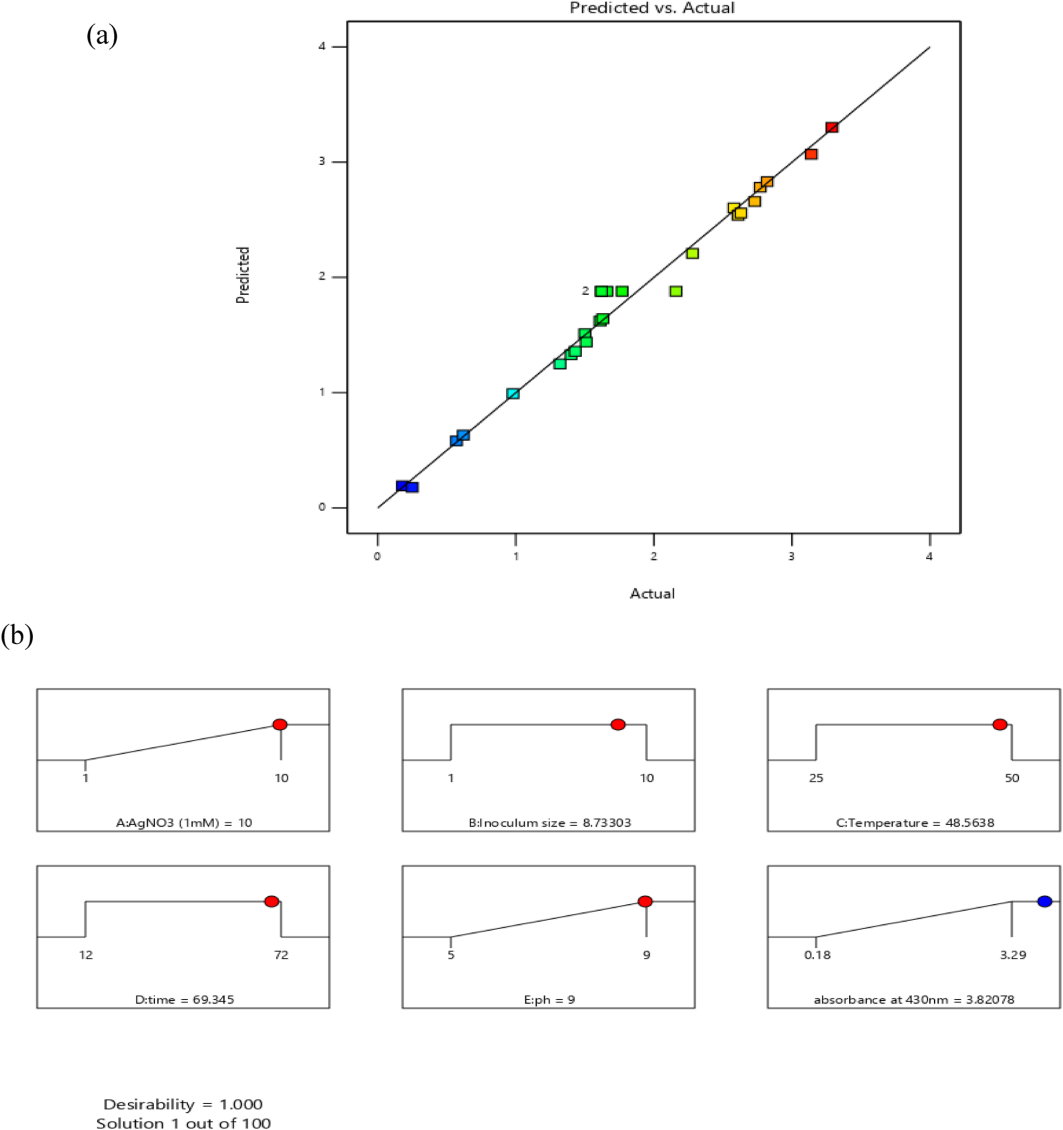
(**a**) The parity plot suggests the correlation between actual and predicted values of biosynthesized silver nanoparticles. (**b**) Optimized ramps for the maximum biosynthesis of silver nanoparticles obtained from CCD of RSM.

Created on second-order response surface design, the interaction between experimental variables such as AgNO_3_ concentration (A), inoculum size (B), temperature (C), time (D), pH (E), and the response was revealed in the system of three-dimensional surface plots as in (Fig. 7a–j). The outline of the distinct surface elucidated both important and irrelevant effects between independent and dependent experimental aspects. A significant interactive outcome of AgNO_3_ amount and the inoculum quantity on the biosynthesis of silver nanoparticles was detected (Fig. 7a). The interaction of metal concentration and the temperature is exposed in (Fig. 7b). An increase in silver nanoparticle synthesis was observed by an increase in temperature. The effect of metal concentration with time (Fig. 7c) and pH (Fig. 7d) showed that both have a significant effect on silver nanoparticle synthesis. The (Fig. 7e) showed that there is a positive relationship between temperature and inoculum size similarly (Fig. 7f) interprets the correlation of time with inoculum size and pH and inoculum size (Fig. 7g) on the response. A rise or decline in pH from its neutral point produced an increase in the biosynthesis of silver nanoparticles. Similarly, an increase in temperature with time (Fig. 7h) and temperature and pH (Fig. 7i) showed that all factors are interactive and have a substantial effect on the biogenesis of silver nanoparticles. The effect of pH and temperature on response specified proportionality between the two variables. Also, time and pH (Fig. 7j) showed that all these variables meaningfully affected the biosynthesis of silver nanoparticles. The particle behavior gets changed in an acidic and alkaline environment. However, the size of silver nanoparticles decreased with increased pH at given time frame. In alkaline pH, monodispersed, spherical nanoparticles were formed with an increased amount. At elevated pH, the reaction rate was increased which result in nucleation and growth of small-sized silver nanoparticles^36^. At basic pH, there is lower aggregation, good yield, fast growth, monodispersed, and increased particle stability^37^. Similarly, particles get aggregated with increased silver nitrate concentration. Grounded on the second-order polynomial equation the equivalence plot represents the appropriate association between predicted and actual investigational values. The perfect fit of the design and the transformation among projected and definite standards are comparative to the remoteness of data points after the slanting line as in (Fig. 8a).

To authorize the correctness and rationality of CCD model of RSM and for better understanding of response, all trials were completed in triplicate. The ideal parameters for the biosynthesis of silver nanoparticles were AgNO_3_ (1 mM) 10 ml, inoculum size (*Bacillus cereus*) 8.7 ml, temperature 48.5 °C, time 69 h, and pH 9 showed in the arrangement of ramps (Fig. 8b). The forecast and genuine values of CCD design were very near (Fig. 8a) viewing the rationality of the applied design.

Gram-positive bacterial strains *Staphylococcus epidermidis, Staphylococcus aureus*, and gram-negative bacterial strains *Escherichia coli, Salmonella enterica, Porteus mirabilis* were used to find the antimicrobial efficiency of biosynthesized silver nanoparticles. Zones of inhibitions (mm) of biosynthesized silver nanoparticles were resolute by using the disc diffusion method. Streptomycin (200 µg/ml) antibiotic was used as a standard against all the five bacterial strains (Table 5). The minimum inhibitory concentrations (MIC) and then minimum lethal concentrations (MLC) were performed by the broth macro dilution method. All the experiments were carried out in triplicate and average ± SD was represented (Table 6). Silver nanoparticles may interact with proteins of bacterial cell membranes and DNA of bacterial cells and cause the membrane disruption. An increase in cell absorptivity and DNA impairment causes cell death and inhibition of bacterial growth^38^. So, we can conclude that the antibacterial action of silver nanoparticles depends on the composition of the bacterial cell wall and cell membrane. Another proposed mechanism revealed that silver nanoparticles get committed to the bacterial cell membrane. As a result of, membrane permeability and respiratory functions get disturbed. The nanoparticles may also penetrate inside the bacterial cell^39^. Small-sized silver nanoparticles would have enhanced the bactericidal effect due to their large surface area as compared to large-sized nanoparticles. Other investigations suggest that positive charge silver ions develop electrostatic attraction with a negatively charged microbial membrane^40^. It was discovered that silver ions from silver nanoparticles were accountable for antimicrobial efficacy^41^. From this we can say that silver nanoparticles can be employed to progress new anti-microbials generations, drug delivery systems, nanomedicines, and many other proposals like silver-based coverings, silver layered medical devices, nano lotions, and nanogels.

**Table 5:** Diameter in mm of zones of inhibition of biosynthesized silver nanoparticles against a standard antibacterial agent streptomycin by disc diffusion method.

Tested strain	Streptomycin (200 μg/ml)	Silver nanoparticles (200 μg/ml) (mean ± SD)	P-value
*Staphylococcus epidermidis*	25	32.12 ± 0.55	0.0421*
*Staphylococcus aureus*	34	38.25 ± 0.05	0.0187*
*Escherichia coli*	30	33.05 ± 1.33	0.0213*
*Salmonella enterica*	27	30.73 ± 0.25	0.0512*
*Porteus mirabilis*	33	35.44 ± 1.08	0.0317*

Data is mean of three replicates ± SD.

*P-value significant ˂ 0.05.

**Table 6:** Broth macro dilution method for determination of minimum inhibitory and minimum lethal concentration values of the biosynthesized silver nanoparticles**.**

Tested strain	MIC (μg/ml)	MLC (μg/ml)
*Staphylococcus epidermidis*	250	400
*Staphylococcus aureus*	200	240
*Escherichia coli*	330	520
*Salmonella enterica*	220	270
*Porteus mirabilis*	140	350

**Figure 9:**
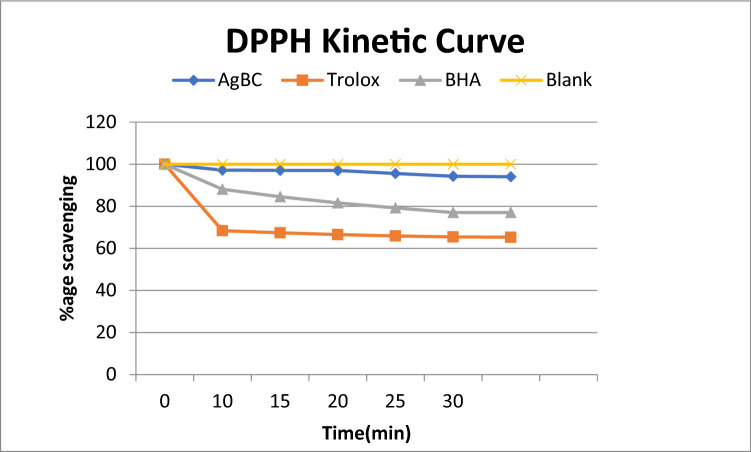
DPPH kinetic curve of biosynthesized silver nanoparticles at different time intervals when TROLOX solution was used as positive control.

**Figure 10:**
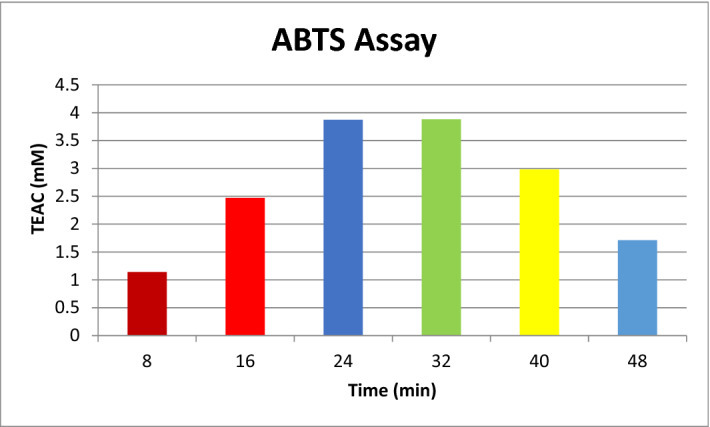
ABTS assay of biosynthesized silver nanoparticles at different time intervals. TEAC stands for Trolox equivalent antioxidant capacity and it represents the antioxidant potential of Trolox solution with equivalent concentration of sample under investigation.

Antioxidants protect the cells against free radicals. An antioxidant stops oxidation by neutralization of produced free radicals as a result of which itself it undergoes oxidation. In the present study, the antioxidant activity of biosynthesized silver nanoparticles was investigated by 2,2-diphenyl-1-picrylhydrazyl-hydrate (DPPH) and 2,2′-Azino-Bis-3-Ethylbenzothiazoline-6-Sulfonic Acid (ABTS) assay. Both of the methods are easy, rapid, sensitive, and produced excellent results of radical scavenging activity for biosynthesized silver nanoparticles^42,43^. DPPH is a commercially available, organic radical having purple color. The purple color faded on reduction showing the antioxidant activity of the sample added. The antioxidant activity of silver nanoparticles at different time intervals was studied and found that 100 μl sample showed a very good radical scavenging activity with time when compared with Trolox and Butylated hydroxyanisole (BHA) standards (Fig. 9). The DPPH free radical scavenging activity was represented by the percentage DPPH remaining. It showed significant activity (94.01% remaining) even after 30minutes. ABTS examination was also carried out to calculate the antiradical activity of biosynthesized silver nanoparticles. The outcomes of ABTS assay were articulated in terms of TEAC values (Fig. 10). TEAC value is a measure of the antioxidant activity of a substance. Higher the value of TEAC greater the antioxidant potential of the substance. The percentage inhibition increases with time and found maximum from 16 to 40 min. TEAC value is positively associated with ABTS cation radical scavenging capacity when Trolox acts as a standard antioxidant compound.

**Figure 11:**
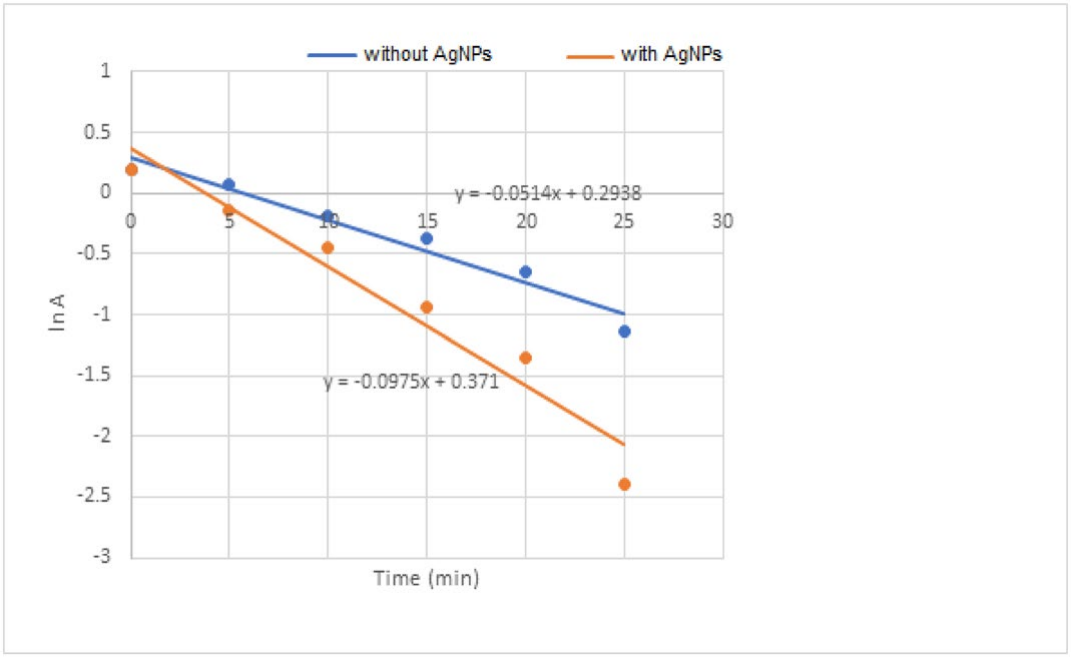
Dye degradation with and without silver nanoparticles.

Methyl orange (MO) is a commonly used water-soluble azo-dye which is widely used in textile, paper, and printing industries. Mostly discharged in the industrial wastewater. Degradation and removal are an exciting area of research. It is orange-red in color in aqueous solution and shows maximum absorbance at 460 nm^44^. Although NaBH_4_ is a robust reducing agent, however, is incapable to reduce methyl orange (MO) successfully. There is a great difference of redox potential of both so the reduction is thermally permitted but kinetically not permitted^45^. The reaction kinetics was studied spectrophotometrically. The reaction follows pseudo-first-order kinetics as the amount of sodium borohydride used is higher and practically remains constant. Only the concentration of methyl orange changes with time. A smooth plot of lnA with time was attained for the degradation of methyl orange with and without silver nanoparticles (Fig. 11). In this redox process, there is considered to be an electron transfer reaction involve between dye and sodium borohydride. In this reaction, sodium borohydride acts as an electron donor and the methyl orange act as electron acceptor^46^. The reduction potential of biosynthesized silver nanoparticles is somewhat between the methyl orange and borohydride ions make the transfer of electrons easier. Due to the large surface area of biosynthesized silver nanoparticles, the rate of reduction processes is enhanced. Both the reactants get adsorbed on the surface of silver nanoparticles. The borohydride ions release electrons to the catalyst surface from which methyl orange gain electrons and get reduced. So the rate of reaction is enhanced in presence of silver nanoparticles^47^. The concentration of methyl orange decrease with time as in Table 7. The rate constant for degradation of methyl orange without silver nanoparticles is calculated to be 0.0514 min^−1^ and with silver nanoparticles is 0.0976 min^−1^. This shows that rate of reaction increases due to silver nanoparticles which confirms its catalytic activity in this reaction.

**Table 7:** Degradation of methyl orange (MO) with and without biosynthesized silver nanoparticles at 460 nm along with lnA calculated from pseudo-first-order kinetics.

Time (min)	Absorbance of MO with AgNPs	lnA	Absorbance of MO without AgNPs	lnA
0	1.21	0.19	1.21	0.19
5	0.87	− 0.14	1.07	0.07
10	0.64	− 0.45	0.83	− 0.19
15	0.39	− 0.94	0.69	− 0.37
20	0.26	− 1.35	0.52	− 0.65
25	0.09	− 2.4	0.32	− 1.14

## Materials and methods

Analytical grade silver nitrate AgNO_3_ (99.95%), nutrient broth, nutrient agar, and all reagents used in this investigation were purchased from Sigma Aldrich Chemicals Germany. Double distilled deionized water was used during this investigation. The bacterial strain *Bacillus cereus* was isolated from contaminated soil samples, purified, and identified morphologically and microbiologically. These attained pure cultures were reserved in nutrient broth agar medium slants and were re-cultured after intervals to synchronize their viability in the laboratory during the study period. For antimicrobial activity, microbes were provided by Mayo Hospital Lahore including *Staphylococcus epidermidis, Staphylococcus aureus*, *Escherichia coli, Salmonella enterica* and*, Porteus mirabilis.*

### Inoculum size preparation

Conical flask of 250 ml comprising 1.5 g of nutrient broth per 100 ml of a buffer (pH 5 to 9) solution prepared in double-distilled deionized water was autoclaved at 121 °C for 15 min. Freshly prepared buffer solutions of pH 5 to 9 were used and their pH was monitored by pH meter. A loop full of *Bacillus cereus* was then inoculated into the autoclaved flask and positioned in an orbital shaking incubator at 37 °C, and 120 rpm for 24 h.

### Conditions optimization for synthesis of silver nanoparticles by RSM

Initial experiments displayed that temperature (°C), time (h), pH (Mole/L of H ions), inoculum size (ml), and metallic concentration (1 mM) are the experimental features that affect the synthesis of nanoparticles. Individual and interactive effects of these factors were calculated by using central composite design (CCD) of response surface methodology (RSM)^48^. To get the set of optimized features for the biosynthesis of nanoparticles, the levels and symbols of these experimental variables are shown in Table 1. Optimization of the procedure was carried out by using Design-Expert ver-12 software (Stat Ease, Inc. MN, USA) formed on complete factorial central composite design with 26 runs as shown in (Table 2). The importance of these constraints on the retort (absorbance at 430 nm) was checked for 5 days. Trials were taken in triplicate, examined on a UV–visible spectrophotometer, and absorbance of every trial at 430 nm was reserved as a response (Y). The results of the central composite design (CCD) trials were analyzed through RSM by means of second-order polynomial equation^49^ as shown below:

$$ {\text{Y }} = {\text{ b}}_{0} + \sum^{{\text{n}}} {\text{b}}_{{\text{i}}} {\text{X}}_{{\text{i}}} + \, \sum^{{{\text{n}} - {1}}} \sum^{{\text{n}}} {\text{b}}_{{{\text{ij}}}} {\text{X}}_{{\text{i}}} {\text{X}}_{{\text{j}}} + \, \sum^{{\text{n}}} {\text{b}}_{{{\text{ii}}}} {\text{X}}_{{\text{i}}}^{{2}} + \, \varepsilon $$

where, Y = response variable, X_i_ and X_j_ = both are independent variables, b_0_ = the coefficient constant, b_i_ = the linear effect coefficient, b_ij_ = the interaction effect coefficient, b_ii_ = the quadratic effect coefficient, ε = random error, n = numeral of variables.

These were articulated in relation to three-dimensional plots viewing the distinct and interactive possessions of these investigational variables on the response. The design was spotted by F-values, lack of fit, and R^2^ values and then the model was recognized. Finally, the full quadratic model was reflected through the analysis of variance (ANOVA). ANOVA outcomes and fit statistics for the quadratic model are briefly presented in Tables 3 and 4.

### Green synthesis of silver nanoparticles

During the green biogenic formulation of silver nanoparticles, 1 ml to 10 ml of silver nitrate (1 mM) was added into 1 ml to 10 ml of prepared inoculum of *Bacillus cereus* (pH 5 to 9) in different flasks. The flasks were kept in an incubator shaker at temperatures (25 to 50 °C) and 120 rpm for the specified period of time (12 to 72 h). All the 26 sets of experiments were performed (Table 2). The progress of silver nanoparticle synthesis was checked by using a UV–visible spectrophotometer.

### Antimicrobial activity

The anti-microbial action of biosynthesized silver nanoparticles with the zone of inhibition, the minimum inhibitory concentrations (MIC), and the minimum lethal concentrations (MLC) was conducted for microbes like *Staphylococcus epidermidis, Staphylococcus aureus*, *Escherichia coli, Salmonella enterica* and*, Porteus mirabilis.* The Kirby-Bauer disc diffusion method^50^ was used to govern the zone of inhibitions of biosynthesized silver nanoparticles. The sterilized Petri plates with Mueller Hinton agar were swabbed with bacterial culture. The discs (6 mm) saturated with standard antibiotic Streptomycin (200 μg/ml) and biosynthesized silver nanoparticles (200 μg/ml) were seeded on the upper layer of prepared agar plates. These were incubated at a temperature of 37 °C for a period of 24 h. The outcomes were obtained by measuring the diameter (mm) of the inhibition zone for each bacterial strain (Table 5).

The minimum inhibitory concentration (MIC) is the lowermost effective amount of silver nanoparticles that completely obstructs the 99% growth of tried microorganisms^51^. The MIC determination was carried out by the broth macro dilution method. For this purpose, the Luria Bertain (LB) broth along with two-times consecutive dilutions of silver nanoparticles in a concentration range of 1600 μg/ml to 0.05 μg/ml was used. A positive controller tube with a test microbe in LB broth and a negative controller tube containing only LB broth was also carried out side by side. The MIC was resolute after 24hours of incubation in an orbital shaker at 37 °C and 120 rpm. For MLC determination, a quantity of 25 μl from each clear tube with no visible growth was planted on Mueller–Hinton Agar (MHA) plates and were incubated at a temperature of 37 °C for 24 h (Table 6). The MLC is the lowermost concentration of silver nanoparticles which executes 100% of the microbial population (no growth on MHA plate). The MIC represents the bacteriostatic effects of silver nanoparticles against the tried microbes while the bactericidal effects were represented by MLC.

The consequence of the antimicrobial action of silver nanoparticles was obtained by (ANOVA) analysis of variance software ver 9. The findings were in triplicate and average ± SD values were represented.

### Antioxidant activity

#### Free radical scavenging ability on DPPH

To evaluate the scavenging ability on DPPH, a standard solution was made by dissolving 25 mg/l of DPPH in methanol, and absorbance was regulated at 1.00 ± 0.02 at 515 nm. A sample solution was made by dissolving 3 mg per ml of distilled water. Now to the 2.5 ml of DPPH solution added 100 μl of the sample mixture. The mixture was stirred energetically and allowed to stay in the black for 30 min^52^. Now measure the absorbance at 515 nm after every 5 min interval for 30 min. The scavenging capacity was calculated using the following equivalence,

$$ \% {\text{ DPPH}}_{{{\text{remaining}}}} = \, \left( {{\text{DPPH}}} \right)_{{{\text{t}} = {\text{t}}}} / \, \left( {{\text{DPPH}}} \right)_{{{\text{t}} = 0}} \times { 1}00 $$

where (DPPH)_t=t_ showed absorbance of DPPH solution at a particular time and (DPPH)_t=0_ showed absorbance of DPPH solution at the starting point.

### Antioxidant activity evaluated by ABTS

For ABTS assay, 7 mM ABTS was formulated in doubly deionized water. To generate ABTS radical 2.45 mM potassium persulfate was added and permitted to stand for 12–16 h in dark. After that, a buffer of pH 7.4 was added and absorbance was kept at 0.70 ± 0.02 at 734 nm. 10 μl (1 mg/ml) of silver nanoparticles were added to 2.99 ml of the above ABTS solution then observed the alteration in absorbance after every 8 min^53^. All absorbance values were taken in triplicate and mean values were calculated. A blank was run in parallel. Percentage inhibition was intended by means of the formula,

$$ {\text{Inhibition percentage}}_{{({\text{at 734nm}})}} = { 1 }{-}{\text{ A}}_{{{\text{sample}}}} /{\text{ A}}_{{{\text{blank}}}} \times {1}00 $$

### Catalytic activity

To study the catalytic degradation of methyl orange (MO), a 2 ml (0.01 × 10^–2^) solution was taken in a cuvette (1 cm path length). In it was added 0.5 ml (0.006 M) solution of NaBH_4_ followed by 0.5 ml (0.050 mg/ml) of biosynthesized silver nanoparticles. The change in the concentration of methyl orange with time was studied spectrophotometrically. The change in absorbance at 460 nm was noted after every 5minutes interval at room temperature. A blank experiment (without AgNPs) was also carried out.

### Ethical approval

The article does not comprise any studies with humans or animals made by any of the authors.

## Conclusion

The biogenic formation of silver nanoparticles was achieved by using *Bacillus cereus* also the process of synthesis was improved by using response surface methodology (RSM). The practice of central composite design (CCD) of response surface methodology (RSM) looked like an important tool to elucidate the discrete and reciprocated effects of numerous investigational data points and their optimization for better biosynthesis of silver nanoparticles in a lucrative and time-effective mode. Classical method of optimizing one factor at a time by keeping other factors constant was avoided as it may cause errors and more time-consuming. For the first time, the biosynthetic conditions of silver nanoparticles through *Bacillus cereus* were optimized through the central composite design of response surface methodology. After performing the designed experiments, the optimized factors were, amount of AgNO_3_ (1 mM) = 10 ml, inoculum size (*Bacillus cereus*) = 8.7 ml, temperature = 48.5 °C, time = 69 h, and pH = 9. The produced silver nanoparticles were small sized ranging from 5 to 7.06 nm with large surface area of 358.78 m^2^/g. The produced nanoparticles were characterized by UV–visible spectrophotometer, FTIR, particle size analyzer, XRD, and SEM. The produced particles were spherical with a large surface area. The antibacterial action of biogenic nanoparticles was evaluated compared to *Staphylococcus epidermidis, Staphylococcus aureus*, *Escherichia coli, Salmonella enterica, Porteus mirabilis.* The antibacterial effectiveness shows an assurance for their use as a robust mediator to treat multidrug-resistant microbes. The biosynthesized silver nanoparticles were also verified for their antiradical effectiveness and it was evaluated through DPPH and ABTS assay. Degradation of an azo-dye (methyl orange) in presence of biosynthesized silver nanoparticles with time gives excellent results. The biosynthesized silver nanoparticles find their applications in biomedical and industrial wastewater treatment.

## Abbreviations

CCD: Central composite design
RSM: Response surface methodology
NPs: Nanoparticles
AgNPs: Silver nanoparticles
FTIR: Fourier transform infrared spectroscopy
XRD: X-ray diffraction
SEM: Scanning electron microscopy
MIC: Minimum inhibitory concentration
MLC: Minimum lethal concentration
TEAC: Trolox equivalent antioxidant capacity
MO: Methyl orange
